# The use of quantitative pupillometry in patients with pituitary tumors: a technical note

**DOI:** 10.1007/s00701-022-05214-w

**Published:** 2022-04-21

**Authors:** Pavlina Lenga, Martin Jakobs, Jessica Jesser, Philip Dao Trong, Andreas W. Unterberg, Christopher Beynon

**Affiliations:** 1 Department of Neurosurgery, Heidelberg University Hospital, Im Neuenheimer Feld 400, 69120 Heidelberg, Germany; 2 Department of Neuroradiology, Heidelberg University Hospital, Heidelberg, Germany

**Keywords:** Optic chiasm, Pituitary tumor, Pupillometry, Neurological pupil index

## Abstract

**Background:**

Pituitary tumors may cause compression of the optic chiasm, resulting in decreased visual acuity. Therefore, decompression of the optic chiasm is a major goal of surgical treatment in such patients. Quantitative pupillometry has been used in various clinical settings for assessing the optic system but has not been applied in patients with pituitary tumors. This study aimed to evaluate the potential of this technique to improve treatment modalities in patients undergoing surgical resection of pituitary tumors.

**Method:**

Pupillometry using the automated NPi 200® Pupillometer was performed in seven patients who underwent surgical resection of large pituitary tumors at the University of Heidelberg in 2018. The neurological pupil index (NPi) was assessed preoperatively and postoperatively, and correlations with visual acuity and magnetic resonance imaging (MRI) findings regarding optic chiasm compression were determined.

**Results:**

All patients experienced visual disturbance due to a large pituitary tumor. The NPi was < 4.0 in all patients in at least one pupil. Intraoperative MRI demonstrated successful decompression of the optic chiasm in all cases. Postoperatively, the NPi values increased, and this increase was correlated with improved visual acuity.

**Conclusions:**

We found that quantitative pupillometry can detect optic chiasm compression in patients with pituitary tumors. Furthermore, postoperative improvement of NPi values may indicate sufficient decompression of the optic chiasm. Further studies are warranted to substantiate the granularity of this technique to gain valuable information for patients with pituitary tumors who are indicated for surgery.

## Introduction

Compression of the optic chiasm commonly causes visual impairment in patients with pituitary tumors. Approximately two-thirds of patients with non-functioning pituitary macroadenomas present with visual deficits [6] and even complete loss of vision [1]. Pituitary tumors may impact the optic system via compression of the optic chiasm and may also compromise oculomotor nerve function, particularly in cases of cavernous sinus infiltration. Neurosurgical tumor resection, which is typically performed via a transnasal approach, is the mainstay treatment in the majority of cases [9]. Decompression of the optic chiasm to prevent vision loss is a major goal of surgery, even if gross resection of the tumor is not possible. Evaluation of visual acuity is mandatory in patients presenting with pituitary tumors. Furthermore, evaluation of visual acuity following surgery is important to confirm sufficient decompression of the optic chiasm.

Quantitative pupillometry has been increasingly applied in the neurocritical care setting. Abnormal pupillometry findings are associated with increased intracranial pressure in patients with traumatic brain injury, aneurysmal subarachnoid hemorrhage, and intracerebral hemorrhage [2]. Neurological pupil index (NPi) values are > 4.0 in healthy individuals and < 4.0 in critically ill patients [11]. Importantly, previous studies suggest that pupillometry is a more reliable tool than standard clinical evaluation with a flashlight for assessing pupillary function [3]. However, to the best of our knowledge, this technique has not been applied to evaluate optic chiasm decompression in patients with pituitary tumors. This study aimed to evaluate the potential of pupillometry to improve treatment modalities in patients undergoing surgical resection of pituitary tumors.

## Methods and materials

### Patients

Seven patients who underwent surgical resection of a pituitary tumor in 2018 at our institution were enrolled. All patients provided written informed consent to participate in the study. The study was approved by the ethics committee of Heidelberg University and registered under the number (S-788/2021).

#### Inclusion and exclusion criteria

Patients aged ≥ 16 years with radiologically confirmed tumor mass of the pituitary gland who underwent a surgical resection via a transnasal approach at our institution were consecutively enrolled. All patients were treated surgically according to a standardized protocol for pituitary tumors at our institution. Tumors were resected via a microscopic endonasal approach. A preoperative assessment of the NPi was also a prerequisite for enrolment into this study. Patients without a preoperative pupillometry assessment were excluded. Baseline characteristics were retrieved from patients’ medical records.

### Assessments

All patients underwent pupillometry using the NPi 200® Pupillometer (Neuroptics, Laguna Hill, USA). The Pupillometer NPi200 is a handheld infrared device that automatically tracks and analyzes pupil dynamics over a 3-s time period. The NPi algorithm was developed to quantify pupillary reactivity. As previously described, the variables analyzed by this algorithm include pupil size, latency, constriction velocity, and dilation velocity [2]. The NPi was assessed 1 day prior to surgery and 1 day after surgery. In longitudinal studies, the correct selection of the interval over which to assess a parameter is of paramount importance. We decided to perform quantitative pupillometry 1 day after surgery to examine potential immediate changes in the NPi. It is well known that immediately after transsphenoidal pituitary tumor resection, edema and posthemostatic material could be present which may significantly impact the adjacent structures of the visual system such as the optic and the oculomotor nerve [12]. Since automated pupillometry is associated with a low interobserver variability, we did not perform repeated examinations at any given time point [3].

MRI or computed tomography (CT) was performed before surgery, and all patients underwent preoperative endocrinological assessment and clinical evaluation of visual acuity. Intraoperative MRI or CT was performed to evaluate the extent of tumor resection and decompression of the optic chiasm.

## Results

**Fig. 1:**
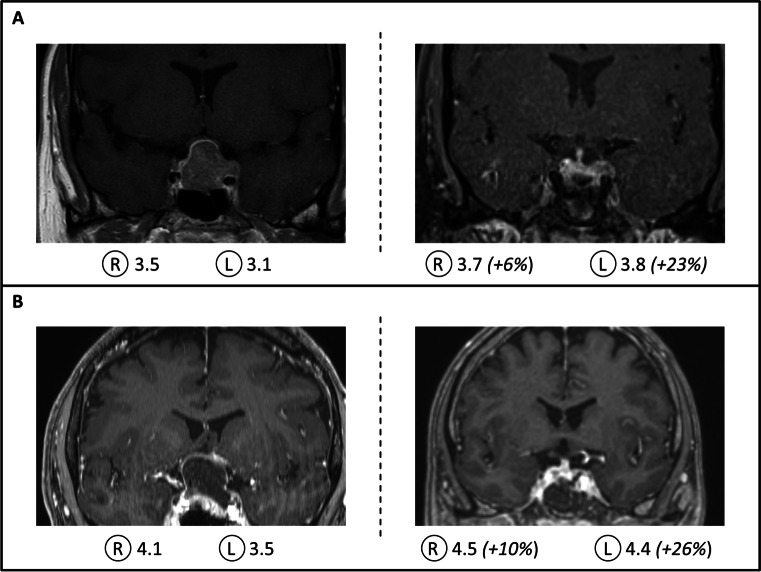
Exemplary results of patients with compression of the optic chiasm. Patient **A** experienced impaired vision due to a solid pituitary adenoma with compression of the optic chiasm on magnetic resonance imaging (MRI) studies (left). The neurological pupil index (NPi) values were < 4.0 (R, right; L, left) in both pupils and improved considerably after surgical tumor resection (right). Intraoperative MRI demonstrated sufficient decompression of the optic chiasm. Patient **B** presented with a cystic pituitary adenoma with compression of the optic chiasm (left). Corresponding to the improved visual acuity after surgery, the NPi values improved, and intraoperative MRI demonstrated sufficient decompression of the optic chiasm (right)

A total of seven patients aged 17 − 66 years were enrolled in this study (Table 1). All patients had impaired visual acuity and imaging-confirmed pituitary tumors with compression of the optic chiasm. The maximum diameter ranged from 11 to 55 mm. Preoperative NPi values were < 4.0 in at least one pupil in all patients (Table 1). Four patients had NPi values of < 4.0 in both pupils. Intraoperative imaging following tumor resection demonstrated decompression of the optic chiasm in all patients (Fig. 1), and gross total tumor resection was achieved in 4 out of 7 patients. Near total tumor resection (due to parasellar growth) was achieved in 2 patients. One patient suffered from extensive parasellar tumor growth, but partial tumor resection resulted in decompression of the optic chiasm. In this patient, histopathological analysis revealed aggressive pituitary adenoma in context of Lynch syndrome [13]. A thorough description of the histological features of the pituitary tumors is displayed in Table 2.

**Table 1:** Overview of preoperative and postoperative patient characteristics

Patient (gender, age)	Clinical symptoms	MRI findings (size: height × width × depth)	Surgery	Histopathology	NPi preoperative		NPi postoperative		Clinical outcome
					Left	Right	Left	Right	
Female 39	Visual impairment	Solid tumor 23 × 27 × 15 mm	Near total resection (ioMRI confirmed)	Pituitary adenoma	3.3	3.5	3.8 (+ 15%)	3.7 (+ 6%)	In hospital: uneventful course, improvement of VA FU (33 months): small recurrence (7 mm), normal VA
Female 63	Visual impairment	Solid tumor 16 × 20 × 14 mm	Gross total resection (ioMRI confirmed)	Pituitary adenoma	3.5	3.2	4.4 (+ 26%)	4.0 (+ 25%)	In hospital: uneventful course, improvement of VA FU (40 months): no recurrence, normal VA
Male 56	Visual impairment Bitemporal hemianopsia	Cystic-solid tumor 31 × 24 × 22 mm	Gross total resection (ioMRI confirmed)	Pituitary adenoma	3.5	4.1	4.4 (+ 26%)	4.5 (+ 10%)	In hospital: uneventful course, improvement of VA FU (4 months): stable, normal VA, Bitemporal hemianopsia (improved)
Female 17	Visual impairment Headache	Solid tumor 21 × 21 19 mm	Near total resection (ioMRI confirmed)	Pituitary adenoma (Prolactinoma)	3.0	3.2	3.8 (+ 26%)	4.0 (+ 25%)	In hospital: uneventful course, improvement of VA FU (38 months): stable tumor remnant, normal VA, Cabergoline therapy
Female 61	Visual impairment	Solid tumor 10 × 15 × 11 mm	Gross resection (ioMRI confirmed)	Pituitary adenoma (GH-secreting)	3.8	4.4	3.9 (+ 3%)	4.1 (− 7%)	In hospital: uneventful course, improvement of VA FU (29 months): no recurrence, normal VA
Female 56	Visual impairment, headaches, double vision	Solid tumor 55 × 31 × 30 mm	Partial resection	Aggressive pituitary adenoma	3.2	3.7	4.8 (+ 50%)	4.8 (+ 30%)	In hospital: uneventful course, improvement of VA Radiation therapy FU (39 months): stable tumor remnant; normal VA
Female 66	Visual impairment, double vision Headache	Cystic tumor 27 × 23 × 21 mm	Gross resection (ioMRI confirmed)	Pituitary adenoma	3.5	4.3	4.6 (+ 31%)	4.6 (+ 7%)	In hospital: uneventful course, improvement of VA FU (3 months): normal VA

*ioMRI*, intraoperative magnetic resonance imaging; *NPI*, neurological pupil index; *GH*, growth hormone

**Table 2:** Histological features of the pituitary tumors

Patient (sex, age [years])	Histopathology	Immunohistochemistry				
		GFAP	AE 1/3	EMA	Synaptophysin	Secreted pituitary hormone
Female 39	Pituitary adenoma	Positive	Positive	Negative	Positive	Prolactin (+ + + +)
Female 63	Pituitary adenoma	Negative	Positive	Negative	Positive	Prolactin (+ +) ACTH ( +)
Male 56	Pituitary adenoma	Negative	Positive	Negative	Positive	HGH (+ +) ACTH ( +) Prolactin ( +)
Female 17	Pituitary adenoma	Negative	Positive	Negative	Positive	Prolactin (+ + + +) HGH (+ +)
Female 61	Pituitary adenoma (GH-secreting)	Negative	Positive	Negative	Positive	HGH (+ + +) Prolactin (+ +)
Female 56	Aggressive pituitary adenoma*	Negative	Positive	Negative	Positive	Prolactin (+ + + +)
Female 66	Pituitary adenoma	Negative	Positive	Negative	Positive	HCG (+ +) Prolactin ( +) LH ( +)

+ : sparse cells positive

+  + : small cell groups positive

+  +  + : larger cell groups positive

+  +  +  + : ≥ 50% of cells positive

^*^Ki-Index up to 40% positive, p53 5% of the cell lines positive indicative for the growth of an aggressive pituitary adenoma

The postoperative assessment revealed NPi improvement in six of seven patients. This improvement ranged from 6 to 50%. In one patient, the NPi value on the right side decreased from 4.4 to 4.1, but visual acuity improved postoperatively.

## Discussion

The deployment of quantitative pupillometry has been established primarily in the neurocritical care setting. A recent study confirmed that anisocoria could be detected by the nursing staff in over 50% of the cases by using the automated pupillometry, whereas the conventional flashlight method showed non-pathological results [3]. Interestingly, the use of quantitative pupillometry in neurocritical care seems to streamline the early detection of intracranial hypertension in patients with traumatic brain injury [7, 13] and can aid in predicting neurological outcomes in comatose patients after cardiac arrest [10]. This emerging technique might present a paramount tool to expediently detect critical cases and help to adequately capture and treat patients with worse prognosis. However, there are only very little data available on the potential of this technique to improve the treatment of patients with impaired visual acuity due to tumor-associated compression of structures of the optic system. To the best of our knowledge, this is the first study evaluating the potential of pupillometry to improve treatment modalities in patients undergoing surgical resection of pituitary tumors. Our findings suggest that automated pupillometry may represent a feasible method to evaluate the mass effects of pituitary tumors on optic system structures.

It is important to accentuate that the assessment of quantitative pupillometry provides valid findings within seconds. Noteworthy, compared to the conventional pupillometry examination with the standard flashlight, the findings are not examiner-dependent. Furthermore, the quantitative pupillometry provides additional information as the constriction and dilation velocity, the pupil size, and the NPi, which is calculated from all of these parameters [3]. Consequently, due to an easy-to-use, affordable, and reliable tool, we were able to show that compression of the neural structures of the optic system due to pituitary adenomas was associated with decreased NPi values. Importantly, surgical decompression of the visual system corresponded with increased NPi values and therefore, this technique may aid in evaluating sufficient decompression of the neural structures of the optic system.

### Review of literature

The use of automated pupillometry in patients with third nerve palsy was recently reported by Kim et al. [8]. A total of 171 patients with third nerve palsy of various etiologies were analyzed, and in 15 patients, a compressive origin due to the presence of a tumor was identified. Abnormal pupillometry findings were highly specific for detecting compressive third nerve palsy and the minimal pupil size showed a significant reduction after recovery. A further study demonstrated that automated pupillometry was equally effective for reversing anisocoria after apraclonidine instillation in the diagnosis of Horner syndrome [14].

In patients with pituitary tumors, decompression of the optic chiasm is the critical pillar for surgical treatment and is associated with an improvement of visual field defects in 80 − 90% of patients [4]. There is an ongoing debate regarding the advantages of endoscopic versus microscopic techniques in the endonasal surgical treatment of pituitary tumors [5]. However, despite advantages regarding intraoperative visualization with the aid of endoscopic techniques, studies have demonstrated that unexpected residual tumor can be detected via intraoperative MRI [16]. Automated pupillometry may present a cost-effective tool in assessing sufficient decompression of the optic chiasm if intraoperative imaging studies are not available.

Furthermore, it is well known that postoperative hemorrhage is a well-recognized complication of transnasal pituitary surgery and occurs in 1–4% of cases. It may cause headache, visual and neurological symptoms, and the acute onset of hypopituitarism. Prompt recognition of these symptoms is vital to identify postoperative hemorrhage and determine whether surgical hematoma evacuation is indicated. Assessment of visual acuity can be difficult in patients with decreased compliance due to the effects of anesthetic drugs. Pupillometry assessment could provide important information for the decision to initiate imaging studies if postoperative hemorrhage is suspected. In patients with intracranial hemorrhage, pupillometry findings were associated with neurological deterioration and therefore, the transfer of this method in this context may prove to be valuable in the detection of postoperative complications.

### Limitations

Although automated pupillometry may represent a valuable tool in the (surgical) treatment of pituitary tumors, our findings must be considered preliminary, and further studies are required before firm conclusions can be drawn. In this study, one patient exhibited a slight NPi decrease in one pupil postoperatively, and this finding was contradictory to the intraoperative imaging findings and improved visual acuity following tumor resection. Thus, other factors may have an impact on the NPi; therefore, the mass effects of pituitary tumors on the optic system and their impact on pupillometry results should be studied in larger patient cohorts. Since there is a lack of clinical data concerning the minimal clinical difference (MCID) between two NPi values, as well as due to the small number of patients included in this technical report, we did not define this important parameter. Standardized preoperative and postoperative visual acuity assessments are needed in order to confirm the validity of NPi results with regard to improvement of visual acuity.

### Conclusions

Our initial experiences with the use of quantitative pupillometry in patients with pituitary tumors demonstrate that it may represent a valuable tool in the assessment of visual disturbances in such patients. The findings may indicate compression of the optic chiasm, and immediate improvements in pupillometry findings following surgery were suggestive of sufficient decompression of the optic chiasm. Further studies are necessary to evaluate the potential of this technique to improve treatment modalities in patients undergoing surgical resection of pituitary tumors.
